# A Bayesian Model to Analyze the Association of Rheumatoid Arthritis With Risk Factors and Their Interactions

**DOI:** 10.3389/fpubh.2021.693830

**Published:** 2021-08-16

**Authors:** Leon Lufkin, Marko Budišić, Sumona Mondal, Shantanu Sur

**Affiliations:** ^1^The Clarkson School, Clarkson University, Potsdam, NY, United States; ^2^Department of Mathematics, Clarkson University, Potsdam, NY, United States; ^3^Department of Biology, Clarkson University, Potsdam, NY, United States

**Keywords:** rheumatoid arthritis, comorbidities, interactions, prediction, Bayesian, NHANES, genetic algorithm, factor analysis of mixed data

## Abstract

Rheumatoid arthritis (RA) is a chronic autoimmune disorder that commonly manifests as destructive joint inflammation but also affects multiple other organ systems. The pathogenesis of RA is complex where a variety of factors including comorbidities, demographic, and socioeconomic variables are known to associate with RA and influence the progress of the disease. In this work, we used a Bayesian logistic regression model to quantitatively assess how these factors influence the risk of RA, individually and through their interactions. Using cross-sectional data from the National Health and Nutrition Examination Survey (NHANES), a set of 11 well-known RA risk factors such as age, gender, ethnicity, body mass index (BMI), and depression were selected to predict RA. We considered up to third-order interactions between the risk factors and implemented factor analysis of mixed data (FAMD) to account for both the continuous and categorical natures of these variables. The model was further optimized over the area under the receiver operating characteristic curve (AUC) using a genetic algorithm (GA) with the optimal predictive model having a smoothed AUC of 0.826 (95% CI: 0.801–0.850) on a validation dataset and 0.805 (95% CI: 0.781–0.829) on a holdout test dataset. Apart from corroborating the influence of individual risk factors on RA, our model identified a strong association of RA with multiple second- and third-order interactions, many of which involve age or BMI as one of the factors. This observation suggests a potential role of risk-factor interactions in RA disease mechanism. Furthermore, our findings on the contribution of RA risk factors and their interactions to disease prediction could be useful in developing strategies for early diagnosis of RA.

## 1. Introduction

Rheumatoid arthritis (RA) is a systemic autoimmune disorder of the joints and internal organs that affects 0.5–1.0% of the adult population worldwide (1, 2). It is a major cause of disability and is associated with an increased risk of premature death (3). The chronic and progressive nature of RA poses a significant financial burden, with the annual societal cost of RA estimated to be $19.3 billion in the United States alone (4). Despite its profound impact on society and the healthcare system, many aspects of this complex, multifactorial disease remain unknown. A variety of genetic, environmental, and behavioral risk factors have been identified for RA and its association with a number of comorbidities has been reported (5). Since current medicine does not offer a cure for RA, the major therapeutic goal is preventing flare-ups, inducing fast remission, and slowing down progressive changes such as irreversible joint deformity (6). Despite RA's demand for close and specialized medical supervision, the number of rheumatologists across the United States has been steadily decreasing. There were roughly 5,000 practicing rheumatologists in 2015, but this number is projected to decrease to 3,500 by the year 2025 (7). One promising approach to address this increasing disparity in the patient-to-rheumatologist ratio is the development of analytical tools to facilitate early diagnosis and predict disease progression, thus enabling better access to care and improving the plan for managing the disease.

RA has a strong connection to age and sex. Disease onset is most likely between 50 and 75 years of age (5, 8) and females are affected 2–3 times more than males (5). Race and ethnicity are also known to influence RA; for example, a lower rate of remission and increased disease activity are reported in African-Americans relative to whites (9). While the reason for such differences is not completely understood, the presence of a “shared epitope”(SE) that is highly correlated with RA severity and outcome is suggested to underlie the higher incidence of the disease in certain sub-populations (10, 11). Apart from demographic factors, several genetic, environmental, behavioral, and socioeconomic risk factors are identified for RA (5, 12). Increased RA incidence in the presence of a family history with 66% heritability observed among twins suggests a genetic link of RA (13). SE alleles within the major histocompatibility complex are shown to have the strongest association with RA, accounting for up to 40% of total genetic risk (12, 13). Environmental factors that can increase the risk of RA include certain infections such as *Porphyromonas gingivalis* bacteria and Epstein-Barr virus (EBV), where an inappropriate immune response to these microbial agents could trigger autoimmunity (14, 15). Additionally, air pollution and occupational exposure to silica have been reported to increase the risk of RA (16, 17). Multiple studies show a strong association of RA with history of tobacco smoking, and the risk of RA increases with the intensity of smoking (18–20). Lower socioeconomic status and less education pose a higher risk of developing the disease (21) as well as experiencing a poorer prognosis (22).

Comorbidities are widespread with RA and often contribute to worse health outcomes (23, 24). Consistent with the complex, systemic nature of RA, these comorbidities often also affect many systems in the body. Among them are widely prevalent chronic conditions such as cardiovascular disease (CVD) and diabetes, which increase the risk of mortality in RA patients (25, 26). Likewise, hypertension and depression increase the risk of disability (26). Gout, another disease of joints, has been found to have a higher association with RA (27). Additionally, RA interferes with the antinociceptive pathway, resulting in enhanced pain perception and leading to a greater risk of sleep problems (28, 29). Several of RA's comorbidities, such as obesity and depression, demonstrate a bidirectional association with RA, implying their presence elevates the risk of developing RA (30, 31). It is of great clinical interest for physicians and researchers to study the concurrent presence of high Body Mass Index (BMI), depression, and CVD in RA patients as it poses a unique clinical repertoire and has significant consequences on affected individuals. Therefore, careful consideration of comorbidities is important for clinicians working in rheumatology care.

Studies have aimed to predict the occurrence of common diseases like CVD to provide early diagnosis or risk assessment using data mining, machine learning algorithms, and mathematical modeling (32). While some studies have attempted to predict RA using a similar approach (33, 34), these studies were neither very selective in defining relevant factors for disease prediction nor did consider their interactions. Karlson et. al. (35) developed prediction models for RA from a combination of clinical and genetic predictors. The models considered age, sex, and smoking as clinical risk factors and studied eight human leukocyte antigen (HLA) and 14 single nucleotide polymorphism (SNP) alleles associated with seropositive RA as genetic risk factors. Models considering either clinical risk factors alone or both clinical and genetic risk factors were compared for discrimination ability using the receiver operating characteristic (ROC) curve. The models with clinical risk factors alone had areas under the ROC curve (AUC) of 0.566-0.626, while models considering both clinical and genetic risk factors had AUC of 0.660-0.752, indicating an improvement of discrimination ability following the inclusion of genetic risk factors. Chibnik et. al. (36) developed a weighted Genetic Risk Score (GRS) from 39 alleles associated with an increased risk of RA. After controlling for age and smoking, the authors used the Genetic Risk Score in a logistic regression to discriminate between non-RA and four phenotypes of RA in the NHS dataset. Their model predicted seronegative, seropositive, erosive and seropositive, and erosive RA with AUCs of 0.563, 0.654, 0.644, and 0.712, respectively. Several other studies (37–40) have performed similar predictive analyses using a combination of environmental and genetic risk factors to create models with good discrimination abilities. The best predictive model we are aware of (as measured by AUC) was developed by Scott, et. al. (41). In this study, the authors considered age, sex, and 25 human leukocyte antigens and 31 single nucleotide polymorphism alleles to develop a model with an AUC of 0.857 (95% CI: 0.804–0.910), indicating high discrimination ability.

**Figure 1:**
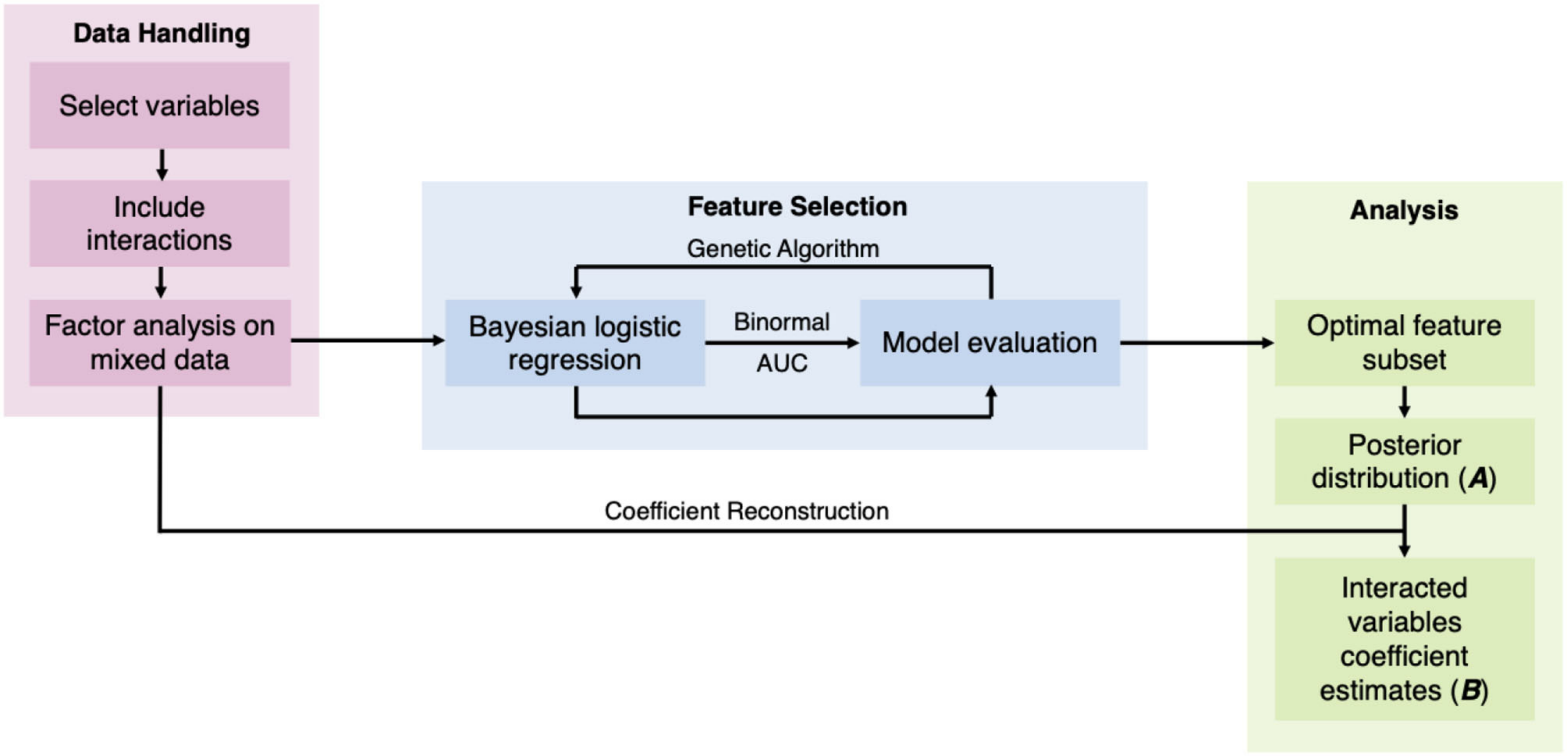
Study methods diagram.

While previous studies have demonstrated the feasibility of predicting RA from environmental and genetic information, patient genetic data are not readily available in a regular healthcare set-up, thus limiting their practical applicability. In this work, we aimed to develop a predictive model of RA using information commonly available in peripheral health centers or rural infrastructures, such as comorbidities, demographic, socioeconomic, and behavioral factors that are known to associate with RA. We used Bayesian logistic regression to build our model and considered up to third-order interaction between the variables (Figure 1). Furthermore, to reduce the computational need without compromising predictive accuracy, we implemented FAMD and wrapper methods, which allowed the selection of the most important variables for the model.

## 2. Materials and Methods

### 2.1. Description of Data and Preprocessing

Subjects in this study were participants in the National Health and Nutrition Examination Survey (NHANES)^1^, a biannual survey designed to assess the health of the US population administered by the Centers for Disease Control and Prevention. NHANES offers freely accessible detailed health datasets on a sample drawn from the US that is representative of the national population. These datasets provide information on demographic variables, socioeconomic condition, survey questionnaires, and bio-specimen examinations. Participants are deidentified and represented by a unique sequence number in each dataset.

**Figure 2:**
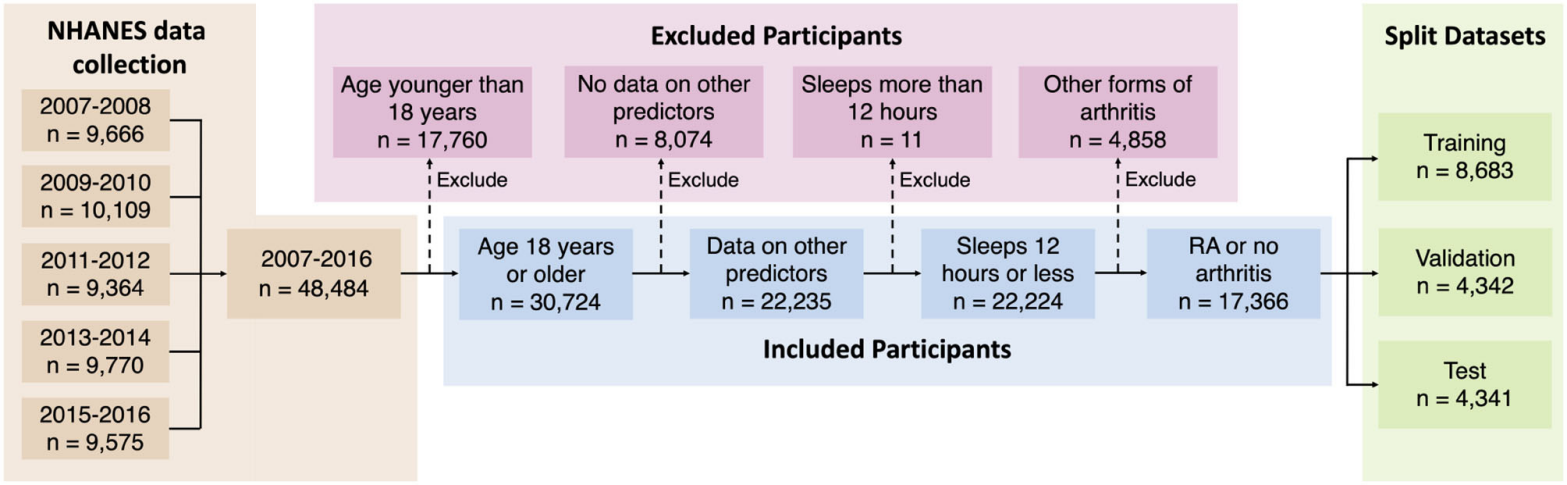
Selection of study population.

NHANES data cohorts from 2007 to 2016 were used in this study, providing an initial dataset with 48,484 participants (Figure 2). The survey protocol and data collection methods for the data were approved by the National Center for Health Statistics Research Ethics Review Board (protocol #2005-06 and protocol #2011-17). The Institutional Review Board (IRB) at the researchers' institution does not require an IRB approval or an exemption for the analysis of de-identified and publicly available NHANES data. NHANES uses a multistage, probabilistic sampling design to select participants and provides sample weights for variables to obtain a more accurate estimate of the nationally representative population. While the implementation of sample weights for complex survey data is straightforward for classical analysis, this is a challenging problem for a Bayesian model and is still an active area of research (42, 43). In our preliminary analysis with NHANES RA sample data, we did not find any substantial changes in the distribution of variables after sample weight adjustment and therefore we used the data in our model without further accounting for the sample weights.

Information on demographics, medical conditions, depression, body measures, blood pressure, diabetes, smoking habits, and sleep were obtained from each release cycle, giving a total of 11 variables. Data for gender, age, ethnicity, and socioeconomic condition were obtained from the demographics datasets. Socioeconomic condition was measured using the ratio of a participant's family's income to their poverty threshold (IPR). Participants 17 years old or younger were excluded from the analysis to prevent confounding effects from juvenile RA. Participants were divided into five categories according to their reported ethnicity: Mexican-American (MA), other Hispanic (OH), white, black, and other non-Hispanic (ONH). The ethnicity variable was coded into four new dummy variables using the white ethnicity as the reference category because it contained the largest number of participants. Self-reported diagnoses of RA and gout were obtained from the medical questionnaire dataset. Depression was measured using the nine-question Patient Health Questionnaire (PHQ) (44). Scores on each of the nine questions were manually summed to create a quasi-continuous variable for measuring depression. BMI for each participant was obtained from the body measures dataset as a continuous measurement of obesity. Systolic blood pressure (BP) was calculated from the average of four readings in the blood pressure dataset. Self-reported diagnosis of diabetes were used in this analysis. Borderline diabetes was not considered as diabetes. Participants were included in the smoking category if they indicated smoking of at least 100 cigarettes in their life on the smoking questionnaire. Nightly hours of sleep were recorded in 1-h increments with a maximum of 12 to accommodate for variations in NHANES data collection between 2007–2014 and 2015–2016.

Participants who responded “don't know,” refused to respond, or had missing data for any variable were excluded from this study, retaining 17,366 participants who fulfilled the selection criteria (Figure 2). We created second- and third-order interactions between the independent variables by multiplying the initial variables together (except for sequence number and RA). Interactions created by squaring binary variables and multiplying mutually exclusive binary variables were removed from the dataset. New variables that represent an interaction between two or three initial variables are termed “interacted” variables. Quantitative variables were centered and scaled to have means of zero and standard deviations of one. This dataset was further divided into training, validation, and test datasets by randomly distributing to a 50–25–25% split for use in model building and validation.

### 2.2. Factor Analysis of Mixed Data

The added interacted variables are highly-correlated, posing a problem for regression analysis. Using factor analysis of mixed data (FAMD) (45) new uncorrelated synthetic variables were created, and data projected onto them. FAMD effectively performs principal component analysis (PCA) on quantitative variables and multiple correspondence analysis on qualitative variables. PCA takes in observations of correlated variables and constructs a change of coordinates such that the synthetic output variables are decorrelated. Similarly, multiple correspondence analysis takes in observations of nominal categorical variables and returns a set of decorrelated synthetic variables that represent the underlying structures in the original data. In both cases, the physical interpretability of the created variables is sacrificed to obtain favorable statistical properties, allowing efficient representation of data by a small set of uncorrelated variables.

In FAMD, a new synthetic variable *v* is created by maximizing the criterion

(1) $$\begin{array}{r} \underset{k\in K_{1}}{\sum }r^{2}\left(k,v\right)+\underset{q\in K_{2}}{\sum }\eta^{2}\left(q,v\right), \end{array}$$

where *K*_1_ are qualitative variables, *K*_2_ are continuous variables, *r*^2^ is Pearson's correlation statistic, and η^2^ is the effect size measure from analysis of variance model (46). A complete disjunctive coding was performed on all qualitative variables. This created a pair of indicator variables corresponding to each state of every categorical variable in the dataset, all of which were already boolean variables. This process creates *K*_2_ indicator variables that are only used in FAMD. The original categorical variables were kept as supplementary variables in the dataset (variables that are not used for calculating the synthetic variables but are projected onto them for interpretation), while all remaining quantitative and indicator variables are active variables (used for calculating the synthetic variables).

A decorrelated set of synthetic variables maximizing (Equation 1) can be computed using the singular value decomposition (SVD) of the data matrix ***M***, whose columns correspond variables and rows to observations, that is to the values of those variables for participants. SVD was performed on all active variables, amounting to calculating matrices ***M*** = ***U*Σ*V***^⊤^, used to project the data onto orthogonal axes (synthetic variables). ***U*** is an orthogonal matrix used to calculate the projections of the participants onto the synthetic variables. ***V*** was used to find the projections of the active variables on the new synthetic variables. The projections of categorical variables onto the synthetic variables are determined from their indicator variables. **Σ** is a diagonal matrix containing the singular values, which are in turn square-roots of variance they explain in the dataset so that **Σ**^2^ is the (diagonal) covariance matrix of the synthetic (decorrelated) variables. Synthetic variables corresponding to variances less than one were omitted to maintain low intercorrelation after the validation and test datasets were projected onto them. Due to the properties of SVD, discarding low-variance synthetic variables is known to be the optimal approach, in the sense of Equation (1), to construction of reduced-order representation of the data. FAMD was performed using the package FactoMineR (47) in R 3.6.0.

### 2.3. Statistical Analysis

Bayesian logistic regression was used to predict RA in this study (48). A Bayesian approach was preferred over standard logistic regression because the former provides full posterior information as opposed to point-estimates by the later, and also allows one to incorporate prior information. The model being linear has also an advantage over the common supervised learning algorithms such as random forest by allowing an easier interpretation of predictor effects, important for this study.

Bayesian regression summarizes model coefficients and predictions with probability distributions. The results are frequently reported using the highest density interval (HDI), which is the smallest interval corresponding to a certain probability of the posterior distribution. Here 50% and 99% HDIs were included in the interval plots of the posterior distributions. Variables are ranked in the interval plots based on the posterior probabilities that their coefficients are greater or less than one (when transformed from log-odds to odds scale). If a coefficient's median is greater than one, the probability that it is greater than one is calculated, Pr(β > 1 | *y*). However, if a coefficient's median is less than one, the probability that it is less than one is used, Pr(β < 1 | *y*). Ties between coefficients with equal probabilities of being greater or less than one are broken using the absolute values of the medians of their posterior distributions. A Bayesian approach also allows us to specify prior information about model coefficients using a probability distribution (the prior distribution).

The posterior distribution for Bayesian logistic regression up to a normalizing factor is given by

(2) $$\begin{array}{r} p\left(\vec{\beta }|y_{1},\ldots ,y_{N},X\right)\propto p\left(\vec{\beta }\right)\overset{N}{\underset{i=1}{\prod }}p\left(y_{i}|\vec{\beta },X\right), \end{array}$$

where $p\left(\vec{\beta }\right)$ is the prior and $p\left(y_{i}|\vec{\beta },X\right)$ the likelihood for each data point. The model uses a total of *K* predictors, combined using coefficients $\vec{\beta }={\left(\beta_{k}\right)}_{k=1}^{K}$, and the added intercept term β_0_. The data set ***X*** contains data points ***X***_*i, k*_, where *i* indexes up to a total of *N* participants and *k* the predictors. Binary variables *y*_*i*_ indicate whether the *i*-th participant has RA (if so, *y*_*i*_ = 1, otherwise *y*_*i*_ = 0). Because we are performing Bayesian *logistic* regression, the distribution $p\left(y_{i}|\vec{\beta },X\right)$ is the Bernoulli distribution

(3) $$p(y_{i}|\vec{\beta },X)=\{\begin{array}{ll} p & y_{i}=1\\ 1-p & y_{i}=0 \end{array}$$

where

(4) $$\begin{array}{rc} p & =F\left(\beta_{0}+\overset{K}{\underset{k=1}{\sum }}\beta_{k}X_{i,k}\right) \end{array}$$

is calculated using the standard logistic function *F*(*x*): = [1+exp(−*x*)]^−1^. Each of the coefficients β_0_, β_1_, …  is assigned a uniform prior, weighing all possible values equally. Although uniform densities supported on the entire real line are improper, i.e., they cannot have densities that integrate to one, such a choice of the prior still leads to a valid posterior and is standard in Bayesian analysis.

We implemented Bayesian logistic regression using Stan in R 3.6.0 through the package RStan (49), which uses Hamiltonian Monte Carlo to sample the posterior distribution described by Equation (2). Markov chains were required to have potential scale reduction factors below 1.1 to indicate approximate convergence, imposing a stringent convergence requirement (50).

### 2.4. Predictive Performance and Feature Selection

A wrapper approach to feature selection was implemented in this study to identify the optimal subset of synthetic variables to predict RA. Feature selection is necessary to identify the most relevant predictors from a larger set, and such operation also improves the precision of estimated effects of the selected predictors. A wrapper approach (as opposed to a filter or embedded approach) uses the predictive performance of subsets of synthetic variables to identify the optimal subset. The predictive performance of the regression models in the genetic algorithm (GA, described below) was determined using the area under the receiver operating characteristic curve. Binormal smoothing of the ROC curve is implemented for its robustness in obtaining an unbiased estimate of the model's true discrimination ability (51). This assumes that the distributions of the predicted probabilities of response for the positive and negative cases can be described by a pair of normal distributions, *y*_1_ and *y*_0_, respectively:

$$\begin{array}{l} y_{1}\sim N\left(\mu_{1},\;\sigma_{1}^{2}\right),\;y_{0}\sim N\left(\mu_{0},\;\sigma_{0}^{2}\right). \end{array}$$

In this study, the binormally smoothed AUC is calculated using two parameters:

$$\begin{array}{l} a=\frac{\mu_{1}-\mu_{0}}{\sigma_{1}}\;\text{and }b=\frac{\sigma_{0}}{\sigma_{1}}. \end{array}$$

The AUC is calculated as

(5) $$\begin{array}{r} \text{AUC}=\Phi \left(\frac{a}{\sqrt{1+b^{2}}}\right), \end{array}$$

where Φ is the standard normal cumulative distribution function. Estimates for *a* and *b* are obtained by linear regression to the equation

(6) $$\begin{array}{r} \Phi^{-1}\left(\mathrm{TPR}\right)=a+b\Phi^{-1}\left(\mathrm{FPR}\right), \end{array}$$

where TPR and FPR represent the true positive and false positive rates across all thresholds of classification.

A radial sweep is used to generate confidence bands for the ROC curve to provide optimal coverage (52). Equation (6) is transformed to polar coordinates with center (FPR = 1, TPR = 0) in ROC space. *r* is calculated for values of θ in increments of 0.01 from zero to π/2. Confidence intervals (CIs) for the AUC and for values of *r* are found from 10,000 bootstrapped samples of the predicted probabilities used to generate the ROC curve.

We used a GA in this study to implement a wrapper approach to feature selection. The GA performs an optimization to find the best subset of synthetic variables for predictive performance according to the AUC Equation (5). The GA was parameterized to have a population size of 500 and run for 200 generations. The GA was seeded with variable subsets always containing the first seven synthetic variables and randomly containing the remaining 45 synthetic variables. All computation for the GA was performed using a server from the Clarkson Open Source Institute at Clarkson University with two Intel Xeon E5-2650 processors with 192 gigabytes of usable physical memory. Running the GA on this server took approximately 2 weeks.

Rank selection was used to determine which variable subsets would be selected for genetic transformation to create the next population. The probability that a subset *x* will be selected is given by

(7) $$\begin{array}{r} p\left(x\right)=\frac{1}{n}\left(\min+\left(\max-\min\right)\frac{\text{rank}\left(x\right)}{n-1}\right), \end{array}$$

where *n* is the size of the population. min represents the expected number of times the subset with the poorest predictive ability is selected, while max represents the same for the subset with the best predictive ability, with the constraint that min+max = 2 is imposed (53). rank(*x*) gives the rank of the variable subset within the population such that the best subset has rank *n*. In this study, we set min = 0.7 and max = 1.3 to allow for substantial generational improvement while maintaining sufficient exploration of the search space.

Each variable subset had a probability of 0.8 to be selected for single-point crossover, which was used for its simplicity and performance in GAs (54). Each subset was also subject to a 0.1 probability of being randomly mutated. Elitism was implemented using 5% of the population to maintain high-quality solutions throughout the GA's search. The optimal subset was tested on a holdout set of data to assess for overfitting.

### 2.5. Coefficient Reconstruction

HDIs for the coefficients of the original variables are obtained from the posterior distributions of the optimal subset of synthetic variables. The optimal feature subset of size *n* was fit to the data using eight Markov chains each with 400 samples of the posterior distribution, creating a 3200-row by *n*-column matrix ***A*** of the probability distributions of the coefficients for synthetic variables in the logistic regression model. Columns corresponding to synthetic variables that were omitted were set to zero in ***A***. Probability distributions for the coefficients of the interacted variables ***B*** are calculated from ***V*** according to the equation below.

(8) $$\begin{array}{r} B=AV^{T} \end{array}$$

Estimates for the binary variables in the interacted dataset were obtained from the difference between the estimates for their indicator variables.

## 3. Results

### 3.1. Variable Selection

**Figure 3:**
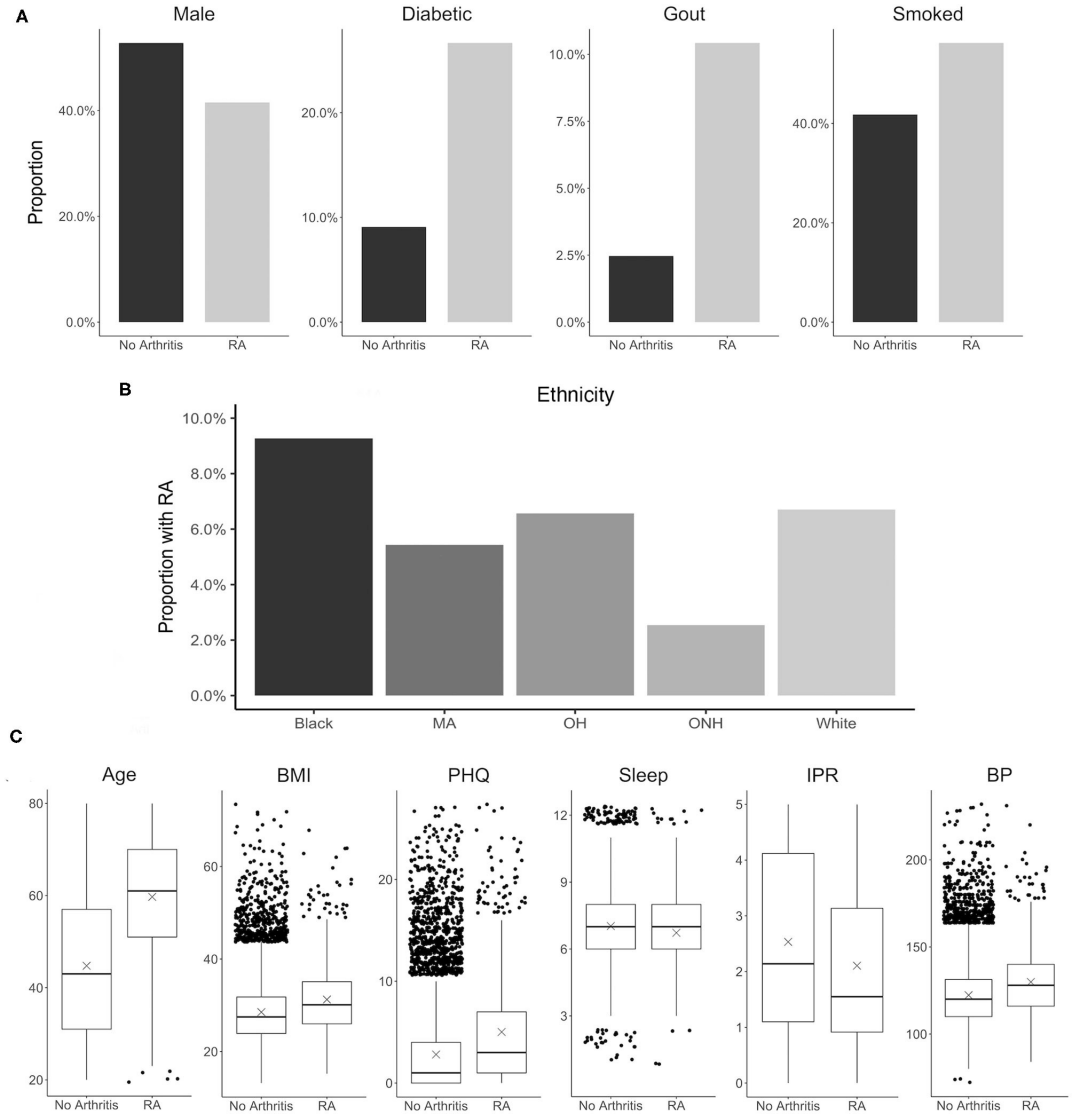
Distribution of various RA risk factors in the study population. **(A)** Comparison between RA and no arthritis population for risk factors coded as binary variables. **(B)** Prevalence of RA among various ethnicity. **(C)** Comparison between RA and no arthritis population for risk factors coded as continuous variables. Units: Age, years; BMI, kg/m^2^; PHQ, PHQ score; Sleep, hours; IPR, nondimensional; Systolic, mmHg. MA, Mexican-American; OH, Other Hispanic.

Selection of risk factor variables to incorporate in our model for RA prediction was guided by their reported association with RA and data availability in the NHANES database. Although a large number of risk factors are reported to be associated with RA, in the present study we selected only a few well-known factors to better understand the contribution of their individual and interaction effects. These variables include disease comorbidities (diabetes, depression, high BMI, hypertension, and gout), demographic factors (gender and ethnicity), socioeconomic factors (IPR), and behavioral factors (smoking and sleep hours) (Figure 3). Other RA risk factors such as asthma or EBV infection were not included in the present analysis even though data for these variables are available in the NHANES database (15, 55). Consistent with the literature, the NHANES dataset demonstrated an association of these risk factors with RA (see Figure 3 and Table 1), although the extent of the difference varied. For example, RA was found to be less common among males (41.4% of RA subjects) but the gender disparity was substantially smaller than reported by previous studies (Figure 3A) (5). This difference could be attributed to the survey-based diagnosis of RA, the data preprocessing procedure, and the inherent design of NHANES (see Supplementary Table 1). Subjects with RA were also more likely to suffer from diabetes, gout, high BMI, depression (measured by PHQ score), and high BP (Figures 3A,C). Risk of RA was found to increase with age, and it was more common among black ethnicity (56) but substantially less prevalent among the ONH population (Figures 3B,C). Behavioral factors such as smoking were observed more among RA subjects, while sleep has a less conspicuous impact even though it was reported previously (29). Interestingly, subjects with RA were found have a lower IPR, suggesting an association of RA with lower economic status.

**Table 1:** Summary characteristics of demographics and risk factors for RA and no arthritis (None) group in the study population.

	**Total participants (***n*** = 17, 366** **)**	
**Prop**.	**RA**	**None**
Male	473 (41.4%)	8,523 (52.5%)
Female	670 (58.6%)	7,700 (47.5%)
Gout	122 (10.7%)	406 (2.50%)
Diabetic	308 (26.9%)	1,480 (9.12%)
Smoked	643 (56.3%)	6,769 (41.7%)
MA	157 (13.7%)	2,652 (16.3%)
OH	120 (10.5%)	1,720 (10.6%)
Black	339 (29.7%)	3,313 (20.4%)
White	476 (41.6%)	6,608 (40.7%)
ONH	51 (4.465%)	1,930 (11.9%)
$\bar{x}$ (***s***)		
Age	59.8 (13.3)	44.8 (16.8)
BMI	31.3 (7.76)	28.6 (6.62)
PHQ	5.04 (5.44)	2.81 (3.91)
Sleep	6.72 (1.75)	7.02 (1.42)
IPR	2.09 (1.49)	2.53 (1.64)
BP	129 (19.8)	122 (17.5)

*Counts and percentages are shown for discrete variables; sample means and standard deviations are shown for continuous variables. Units: Age, years; BMI, kg/m^2^; PHQ, PHQ score; Sleep, hours; IPR, unitless; Systolic, mmHg. MA, Mexican-American; OH, Other Hispanic; ONH, Other Non-Hispanic*.

A total of 11 risk factors were considered in our study, which generated 14 first-order variables including 4 binary variables obtained from dummy coding ethnicity, using the white population as the reference category. For model building and validation, the dataset was further divided into training, validation, and test categories (Table 2). The distribution of the variables were found to be nearly equivalent across each category, indicating an even split after data preprocessing. A slightly greater variation among the three datasets was observed for the RA group, which could be attributed to a substantially smaller number of individuals in this group than the control no arthritis group. In order to analyze second- and third-order interactions, we created 475 interaction variables from the 14 first-order variables, leading to a total of 489 variables.

**Table 2:** Breakdown of participants into training, validation, and test datasets with comparison of summary characteristics between RA and no arthritis (None) groups in each dataset.

**Prop**.	**Training (** ***n*** **= 8,683)**			**Validation (** ***n*** **= 4,342)**			**Test (** ***n*** **= 4,341)**		
	**All**	**RA**	**None**	**All**	**RA**	**None**	**All**	**RA**	**None**
Male	0.523	0.436	0.529	0.513	0.353	0.524	0.523	0.434	0.529
Gout	0.030	0.103	0.024	0.033	0.112	0.028	0.027	0.100	0.022
Diabetic	0.106	0.270	0.095	0.099	0.283	0.087	0.098	0.244	0.088
Smoked	0.428	0.555	0.419	0.419	0.543	0.410	0.435	0.595	0.424
MA	0.163	0.128	0.166	0.157	0.152	0.157	0.163	0.129	0.166
OH	0.105	0.097	0.106	0.114	0.123	0.114	0.102	0.111	0.101
Black	0.208	0.305	0.201	0.216	0.309	0.210	0.205	0.269	0.200
White	0.410	0.427	0.408	0.402	0.368	0.404	0.412	0.448	0.409
ONH	0.114	0.043	0.119	0.111	0.048	0.115	0.118	0.043	0.124
$\bar{x}$ (***s***)									
Age	45.6 (16.9)	59.9 (13.3)	44.6 (16.6)	45.9 (17.1)	59.5 (13.4)	45.0 (16.9)	45.8 (17.1)	59.6 (13.2)	44.8 (16.9)
BMI 28.7 (6.65)	31.4 (7.49)	28.5 (6.54)	28.9 (6.81)	31.6 (7.75)	28.7 (6.70)	28.6 (6.59)	30.6 (7.91)	28.4 (6.47)	
PHQ	2.89 (4.03)	4.80 (5.45)	2.76 (3.88)	3.01 (4.16)	5.37 (5.69)	2.84 (3.98)	3.03 (4.02)	5.11 (5.18)	2.88 (3.88)
Sleep	7.01 (1.44)	6.83 (1.68)	7.02 (1.42)	7.02 (1.47)	6.79 (1.76)	7.03 (1.45)	7.01 (1.44)	6.46 (1.81)	7.05 (1.40)
IPR	2.50 (1.64)	2.13 (1.50)	2.53 (1.64)	2.50 (1.63)	2.05 (1.50)	2.54 (1.63)	2.51 (1.65)	2.11 (1.48)	2.54 (1.65)
BP	123 (17.9)	130 (19.9)	122 (17.6)	123 (18.0)	130 (20.6)	122 (17.7)	123 (18.3)	129 (19.7)	122 (18.1)

*Proportions are shown for binary variables. Sample means and standard deviations are reported for continuous variables. Units: Age, years; BMI, kg/m^2^; PHQ, PHQ score; Sleep, hours; IPR, unitless; Systolic, mmHg. MA, Mexican-American; OH, other Hispanic; ONH, other non-Hispanic*.

### 3.2. Predictive Performance

**Figure 4:**
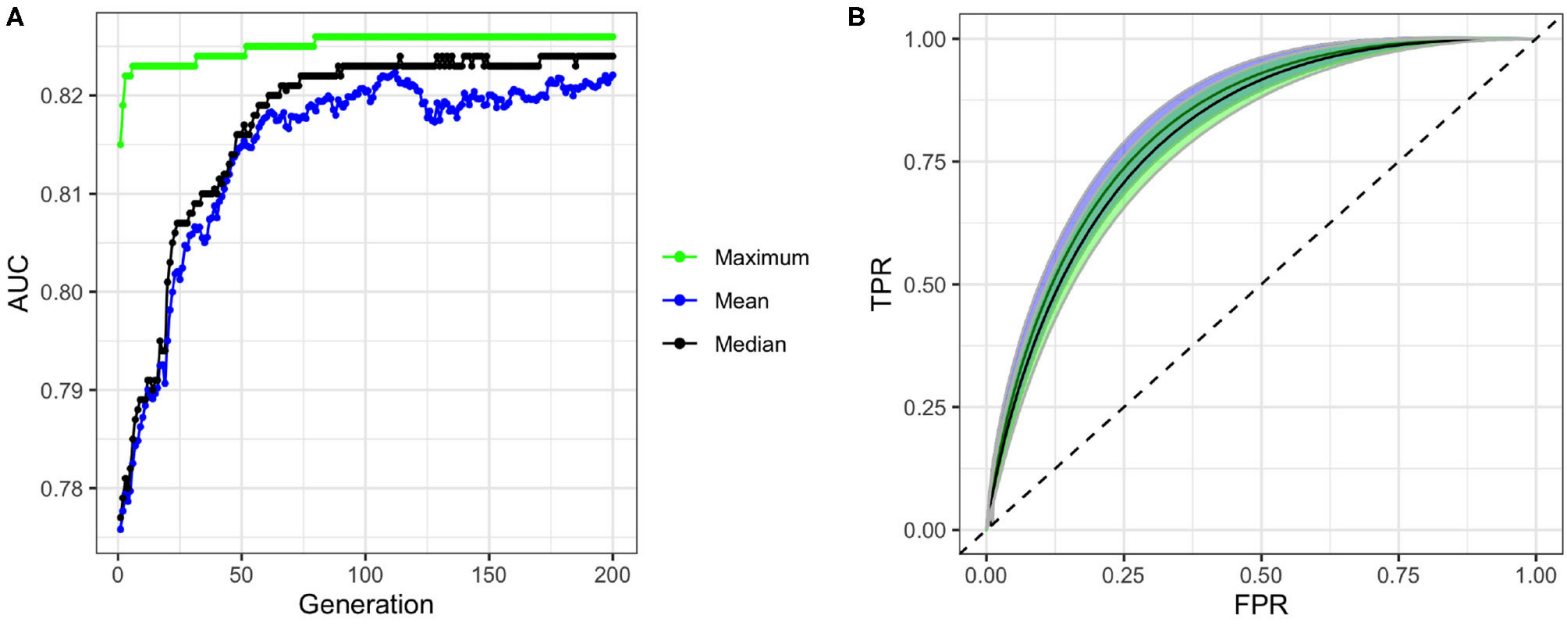
Performance of genetic algorithm (GA) for feature selection. **(A)** Convergence of GA on an optimal subset of synthetic variables with maximum, mean, and median fitness values in each generation of the search. **(B)** ROC curves and confidence bands of optimal model predicting on validation datasets (confidence region shaded blue) and test datasets (confidence region shaded green). Dashed line represents the ROC curve of a model with no predictive ability, corresponding to an AUC of 0.5.

To build our model, we first excluded the highly correlated variables from the total set of variables containing higher-order interactions. Since our data contained both categorical and continuous variables, we implemented FAMD to identify the correlated variables. A total of 52 synthetic variables with variances greater than one were obtained by FAMD that represented 92.3% of the variation in the training data. Table 3 summarizes these synthetic variables according to the percentage of variance explained by each of them. A feature selection from these synthetic variables was further performed by a wrapper approach using GA. An optimal subset containing 33 of these synthetic variables was identified that provides the greatest discrimination ability. Figure 4A shows the progression of the GA's search to find the subset of synthetic variables that best predicts RA. 33 of the 52 total synthetic variables were selected through this process, which was able to predict RA with a smoothed AUC of 0.826 with 95% CI of 0.801–0.850 (Figure 4B). The potential scale reduction factors ($\hat{R}$) and estimated coefficients from the final regression model for these selected synthetic variables are shown in Table 3. For variables omitted through the feature selection process, the medians for posterior distributions of coefficients (β) were set to one and do not have $\hat{R}$ values (Table 3). This subset of variables was also used on the test dataset to obtain a smoothed AUC of 0.805 (95% CI: 0.781–0.829), indicating high accuracy on external data and that the model was not overfitting to the training dataset during regression or the validation dataset during feature selection.

**Table 3:** Percentage of variance explained, $\hat{R}$, and medians for posterior distributions of coefficients for synthetic variables (β) returned by FAMD.

**Var**	**% Exp**	**$\hat{R}$**	****β****	**Var**	**% Exp**	**$\hat{R}$**	****β****	**Var**	**% Exp**	**$\hat{R}$**	****β****	**Var**	**% Exp**	**$\hat{R}$**	****β****
1	10.3	1.00	1.0963	14	1.60	1.00	0.9788	27	0.849	—	1	40	0.442	1.00	0.9086
2	7.30	1.00	1.0545	15	1.59	1.00	0.9239	28	0.821	—	1	41	0.433	—	1
3	6.65	1.00	0.9542	16	1.49	1.00	0.8932	29	0.802	1.00	1.1096	42	0.419	—	1
4	6.49	1.00	0.9640	17	1.24	—	1	30	0.740	1.00	1.0633	43	0.387	—	1
5	6.17	1.00	0.9967	18	1.21	1.00	0.9688	31	0.658	1.00	1.0092	44	0.367	—	1
6	5.96	1.00	0.9963	19	1.18	1.00	0.9598	32	0.630	1.00	0.9839	45	0.343	—	1
7	5.19	1.00	0.9836	20	1.15	1.00	1.0240	33	0.590	1.00	0.9812	46	0.336	—	1
8	4.26	1.00	1.0169	21	1.07	—	1	34	0.552	—	1	47	0.304	1.00	0.9828
9	3.31	1.00	0.8923	22	1.04	—	1	35	0.537	1.00	0.9777	48	0.281	—	1
10	2.91	1.00	1.0987	23	1.02	—	1	36	0.493	1.00	1.0614	49	0.277	1.00	0.9798
11	2.61	1.00	0.9484	24	0.931	—	1	37	0.491	—	1	50	0.262	1.00	1.0314
12	1.83	1.00	1.0846	25	0.903	1.00	1.0062	38	0.470	1.00	0.9688	51	0.248	1.00	0.8923
13	1.61	1.00	1.0362	26	0.872	—	1	39	0.466	—	1	52	0.230	—	1

*Synthetic variables omitted through feature selection have β set to one and do not have $\hat{R}$ values*.

Interestingly, we find that even the first-order variables alone are highly predictive, with an AUC of 0.823, and that higher-order interactions yield only a small improvement of AUC to 0.826. Furthermore, our approach can generate a predictive accuracy higher than most previous works reported even when using a small set of first-order variables (see Supplementary Table 2) (35–37). For example, considering age and smoking alone can generate a model with an AUC of 0.748, and including sex further increased the AUC to 0.772. While these findings suggest the potential of model building from first-order variables alone, future studies are required to identify the set of variables that maximizes the predictive accuracy of the model.

### 3.3. Risk Factor Interactions

**Figure 5:**
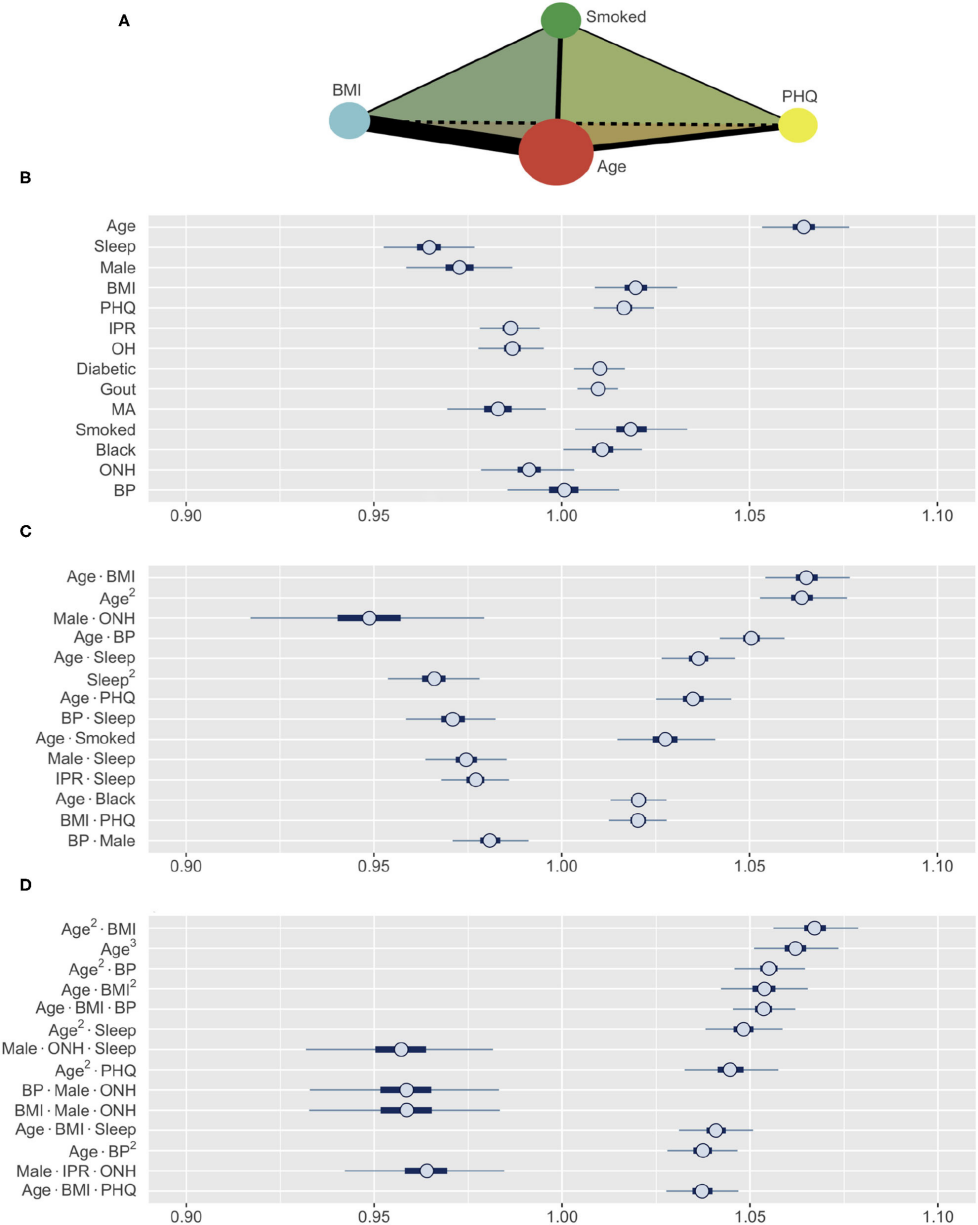
**(A)** A schematic illustrating the interaction analysis between four risk factors. Size of vertices, thickness of edges, and color of faces denote the size of first-, second-, and third-order effects, respectively. **(B–D)** Posterior distributions of standardized coefficients for selected variables. HDIs for **(B)** all first-order variables, **(C)** most influential second-order, and **(D)** third-order variables (inner bounds, 50% HDIs; outer bounds, 99% HDIs). Horizontal scale represents the odds multipliers for risk of RA for one standard deviation increase in value of the variables. MA, Mexican-American; OH, Other Hispanic; ONH, Other Non-Hispanic.

The subset of synthetic variables returned by the GA is not easily interpretable on its own. Each synthetic variable represents a latent variable that is a linear combination of the total pool of 489 variables. The posterior distribution of the synthetic variables obtained through this process was used to construct HDIs for each of the 489 variables using Equation (8). Furthermore, to allow intuitive comparison across variable types and effect orders, the coefficient estimates were computed for the standardized versions of the variables (Figure 5). Thus, the variables with HDIs further away from 1.0 are more significant predictors of RA, while a narrower interval indicates a greater certainty about how a specific variable affects RA. The analysis aims to identify the effects of first-order variables and the influence of any second- and third-order interactions as illustrated in Figure 5A.

The prediction of RA in the test dataset by the first-order variables overall aligns well with the association of these variables to RA observed in Figure 3. Age, BMI, depression (PHQ score), diabetes, gout, and smoking are found to be positive predictors, while male gender and financial wellness (IPR) reduce the risk of having RA (Figure 5B). A clear influence of ethnicity is also observed: Risk of RA is higher among black population and lower among Mexican-American population when compared against white. Interestingly, sleep emerged as a strong negative predictor (the most influential first-order variable after age), even though an association of RA and sleep was not clearly observed in the data. In contrast, systolic BP played no effect on RA prediction as a first-order variable (HDI roughly symmetric about one), although RA subjects had a higher mean systolic BP than the control population. The key first order effects are summarized in Table 4 and RA probabilities against the amplitude of variables are shown by marginal effects plots for a few representative variables in Supplementary Figure 2.

**Table 4:** Summary of key findings for (A) first-order, (B) second-order, and (C) third-order variables.

**(A)**	**Risk factor**	**$\hat{\beta }$**	**Comments**
	Age	1.0533–1.0765	• Aging increases risk for RA
			• Most influential first-order effect
	Sleep	0.9526–0.9768	• More sleep decreases risk of RA
	BMI	1.0088–1.0308	• Higher BMI increases risk of RA
			• Weaker first-order effect
	BP	0.9856–1.0153	• No direct effect on risk of RA
	ONH	0.9785–1.0032	• No direct effect on risk of RA
**(B)**	**Risk factor**	$\hat{\beta }$	**Comments**
	Age^2^	1.0527–1.0752	• Effect of increased age on RA risk is greater at older ages
	Age·BMI	1.0535–1.0770	• Age and BMI have the strongest second-order interaction
	Male·ONH	0.9171–0.9793	• Being male and ONH ethnicity markedly reduces risk for RA
	Age·BP	1.0421–1.0592	• Interaction of age and BP increases risk of RA
	BP·Sleep	0.9585–0.9824	• Higher BP further lowers RA risk afforded by sleep
**(C)**	**Risk factor**	$\hat{\beta }$	**Comments**
	Age^2^·BMI	1.0562–1.0787	• Strong third-order interaction between age and BMI
	Age^3^	1.0511–1.0735	• Effect of increased age on RA risk is greater at older ages
	Age^2^·BP	1.0459–1.0645	• Third-order interaction between age and high BP increases RA risk
	Age·BMI·BP	1.0454–1.0621	• High BP further increases RA risk from aging and high BMI
	Male·ONH·Sleep	0.9319–0.9816	• Sleep adds to lowering RA risk afforded by being male and ONH

*Ninety-nine percent HDIs are shown for estimated regression coefficients on the odds scale*.

Apart from the effects of individual first-order variables, we were interested to identify any influence of higher-order interactions in RA prediction. Figure 5C enumerates the 14 most influential second-order variables observed in our study. Age turns out to not only be the strongest first-order predictor variable but also to have prominent second-order interactions with several other variables, including BMI, BP, depression, sleep, and smoking. The strongest second-order interaction effect was found between age and BMI (median: 1.0648, 99% HDI: 1.0535–1.0770), which is comparable to the influence of age (1.0642, 1.0533–1.0757) or three times the influence of BMI (1.0196, 1.0088–1.0306), considered individually (Table 4). Interestingly, the second-order effect of age (1.0636, 1.0527–1.0752) is similar in magnitude to its first-order effect, suggesting that the effect of age on RA risk increases with age. We also observed several second-order interactions to reduce the risk of RA. For example, the combination of ONH ethnicity with male gender strongly reduces the risk of having RA (0.9485, 0.9156–0.9797), even though ONH does not have a significant influence in lowering RA risk and male gender has a less prominent effect. This finding suggest the second-order interaction with male gender could underlie low RA prevalence observed among ONH ethnicity (Figure 3B). Sleep demonstrates an interesting interaction effect on RA. While increased sleep hours was found to lower the risk of RA, its second-order effect with age increased the risk significantly, suggesting an altered role of sleep on the body's immune system with aging.

Our model was also able to reveal the existence of strong third-order interactions. Figure 5D lists 14 most prominent third-order interactions where we find the frequent appearance of a few variables, with age and BMI being most common. Other factors involved in strong third-order interactions are gender, ONH ethnicity, sleep, depression, and BP. Similar to the second-order interactions, these third-order interactions are seen to either increase or decrease the risk of RA (Figure 5D and Table 4). In particular, for interactions posing high risk, we often observe age and BMI, either as a third-order variant of the interaction between these variables, or in combination with a third variable such as BP, sleep, or depression. By contrast, the coexistence of ONH ethnicity with male gender in a third-order interaction prominently reduces the risk of RA when associated with sleep, BMI, BP, or IPR as the third variable. Thus, variables such as sleep or BP, when involved in third-order interactions, can both increase or decrease the risk of RA, suggesting a complex interplay of underlying physiological mechanisms.

#### 3.3.1. Range of Interactions: Age vs. BMI

**Figure 6:**
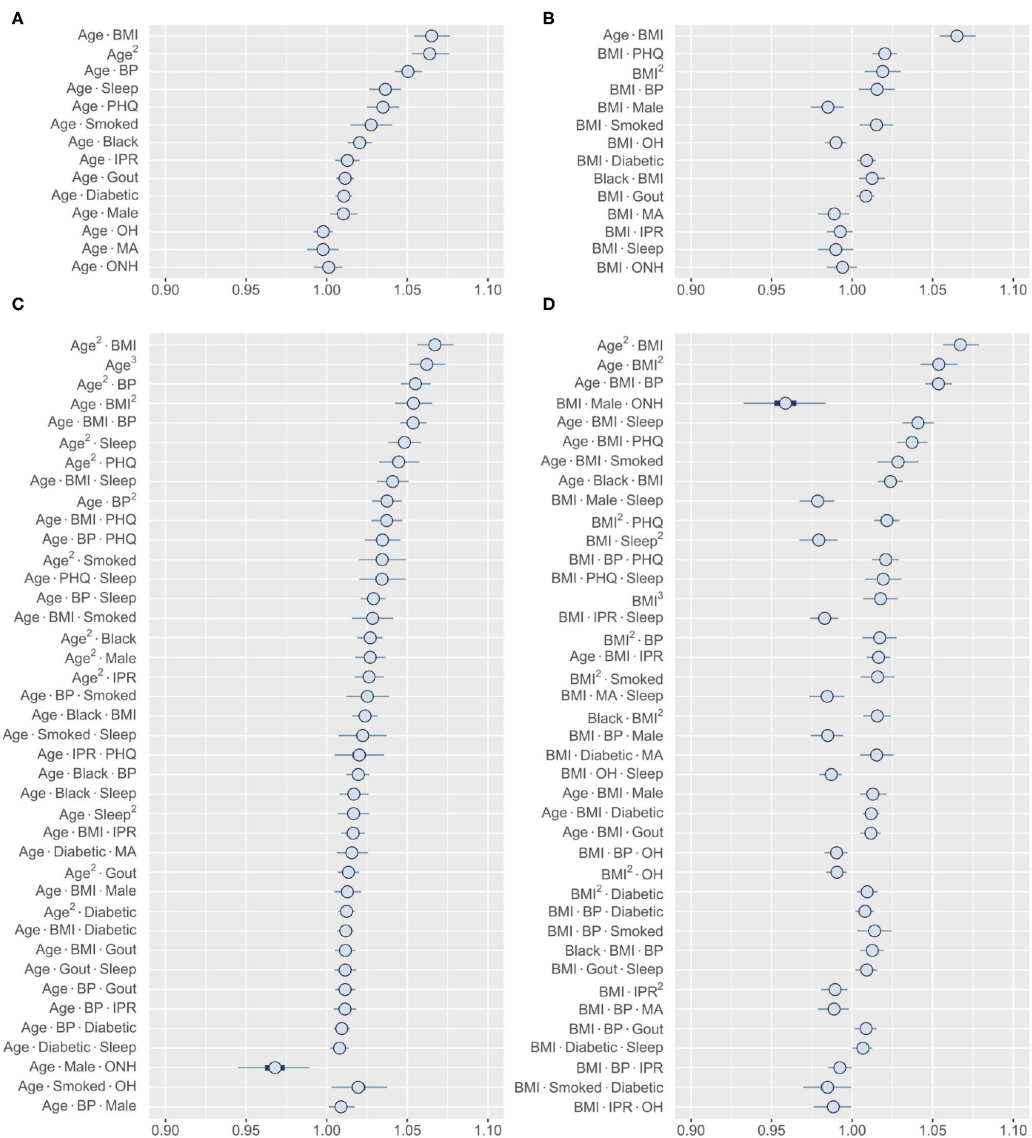
Higher-order interactions of age and BMI. **(A,B)** Posterior distribution of standardized coefficients for all second-order interactions of age **(A)** and BMI **(B)**. **(C,D)** Posterior distribution of standardized coefficients for a selection of 40 third-order interactions involving age **(C)** and BMI **(D)**. Inner bounds and outer bounds represent 50% HDIs and 99% HDIs, respectively. MA, Mexican-American; OH, Other Hispanic; ONH, Other Non-Hispanic. Horizontal scale shows odds multipliers for risk of RA for a one standard deviation increase in value of variable.

The finding of several prominent second- and third-order interactions in our model further motivated us to investigate the range of interactions for an individual risk factor. In this direction, we focused on comparing age and BMI, two variables that demonstrated the strongest higher-order interaction (Figure 6). Our analysis shows that these two variables have very different interaction profiles. Age demonstrates strong second-order interactions with multiple comorbidities (BMI, BP, and depression), sleep, and smoking, all of which increase the risk of RA (Figure 6A). In contrast, second-order interaction effects to BMI are moderate to weak (except with age) and, depending on the interacting variable, increases or decreases the RA risk (Figure 6B). The third-order interactions for age and BMI follow a similar pattern as observed in the second-order interactions, except the combination of male and ONH ethnicity reduces RA risk (Figures 6C,D). We hypothesize that general changes in body physiology accompanied with aging cause other risk factors to have a greater impact on RA, resulting in these interaction effects. In contrast, high BMI potentially elicits specific influence in the pathophysiology of interacting risk factors, increasing or decreasing the magnitude of the effects. Together, these results confirm that the interactions of a risk factor with other risk factors are highly specific in nature and are dependent on the variables considered.

#### 3.3.2. Influence Through Interactions: BP and ONH Ethnicity

**Figure 7:**
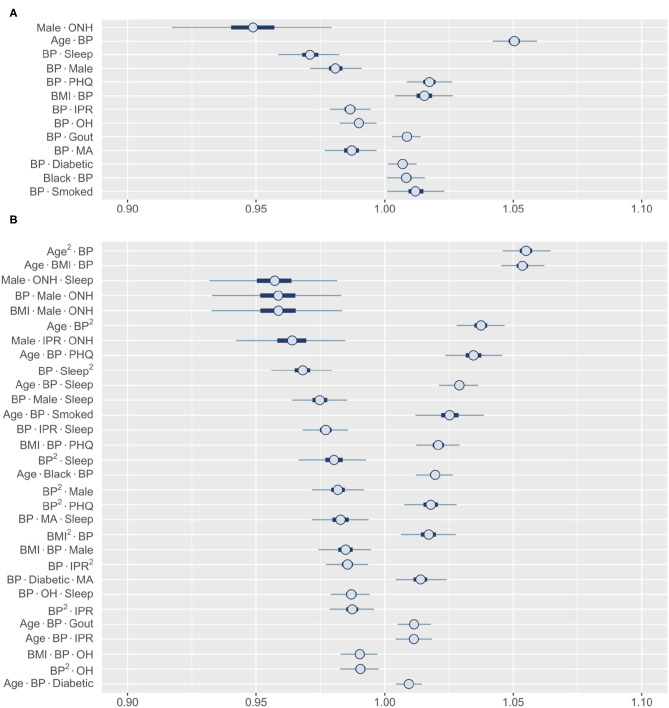
Posterior distributions of most relevant interactions that include either BP or ONH ethnicity as one risk factor. **(A)** 13 most influential second-order interactions and **(B)** 30 most influential third-order interactions are shown. Inner bounds represent 50% HDIs and outer bounds represent 99% HDIs. Standardized coefficient estimates are shown. MA, Mexican-American; OH, Other Hispanic; ONH, Other Non-Hispanic. Horizontal scale shows odds multipliers for risk of RA for a one standard deviation increase in value of variable.

Finally, we wanted to explore the higher-order interactions for risk factors that did not show a significant first-order effect. Among all first-order effects, only BP and ONH category had 99% HDIs that contained one (Figure 5B). Identifying the most influential second-order interactions for BP or ONH category reveals that 12 out of the top 13 involve BP (Figure 7A). The only interaction involving ONH category included in this list (it was also the strongest interaction) is with male gender, strongly lowering the risk for RA. In contrast, the posterior distribution of the interactions of BP indicate that the risk could both increase or decrease depending on the specific interaction. For example, the risk can increase from interaction with age, depression, and BMI, while sleep and male gender reduce the risk. Interestingly, we found the interaction effects of BP with individual risk factors to be similar to their first-order effects. Thus, high BP is expected to enhance the effect of an interaction between risk factors on RA risk. The third-order interactions corroborate well to the second-order interactions with BP occupying 29 of the top 30 interactions (Figure 7B). The effect follows the pattern demonstrated by the interaction between the other two factors. While hypertension is generally considered as a comorbidity of RA, there is a lack of consensus on the true association between RA and hypertension (57). Our finding that BP does not have a significant first-order effect but has prominent interaction effects with coexisting conditions, offers a potential explanation for the varying results reported in the literature.

## 4. Discussion

In this work, we have developed a Bayesian regression model to characterize the risk of RA from common comorbidities, demographic, socioeconomic, and behavioral factors that are known to associate with RA. Apart from providing high predictive accuracy, our model is able to capture the effects of individual variables as well as the important higher-order interactions between them. Consistent with previous literature, known RA risk factors such as depression, high BMI, and smoking are also found to be predictors of RA in our model. Additionally, our model shows that age is not only a key predictor for RA, but also has strong interaction effects with several other variables; prominent among them are BMI, BP, depression, and smoking. Interestingly, some variables such as ONH ethnicity have weak influence as a single-order variable, but their combination with certain other variables (male gender in case of ONH ethnicity) could elicit a prominent higher-order interaction. The knowledge of these strong interactions will help to determine if a person is at a higher or lower risk of RA when both conditions coexist.

One of our primary objectives in this study was to identify and elucidate the effects of important higher-order interactions between risk factors in the prediction of RA. The main challenge in performing such a study comes from the exponential increase in the number of synthetic variables as more higher-order interactions are considered, correspondingly increasing the computational cost. This limitation led us to restrict our study to a maximum of third-order interactions. Our implementation of FAMD further reduced the number of predictor variables analyzed during regression, substantially lowering the requirement for computation. FAMD also allowed the consideration of both categorical and continuous risk factor variables in the model.

In our model, we used feature selection to select an optimal subset of synthetic variables. This step was introduced to not only improve the model's predictive ability but also to obtain a greater precision in determining the effect of risk factors on RA. When studying the manifold interactions between these risk factors, increased precision from feature selection helps to address increases in posterior variances resulting from dramatic increases in the number of variables being analyzed (see Supplementary Figure 1). We implemented a wrapper method for feature selection. However, there are alternative approaches, the most common being filter methods (58). Filter methods employ a ranking system to determine the most relevant variables before any prediction is performed (59), some examples of which include the Pearson correlation coefficient, Fisher score, and mutual information (58). Filter-based approaches generally perform faster than wrapper methods since they do not require the predictive model to be run simultaneously. However, because of this, they do not necessarily return the optimal subset of features for prediction (59). Additionally, some filter methods are prone to selecting redundant features (59), while wrapper methods find the optimal subset based on their performance in the predictive model and do not encounter this issue. Thus, employing a wrapper approach for feature selection allowed us to determine the most important subset of synthetic variables for prediction, and subsequently enabled more precise estimates of the effects of interactions between risk factors on RA. One downside of wrapper methods is that they are generally more computationally expensive than filter methods and implementation of techniques based on exhaustive searches can become computationally infeasible for large datasets (59). To overcome this limitation, we implement a wrapper approach using a GA, a type of evolutionary algorithm, and is capable of providing high-quality solutions with reasonable computational effort (60).

Although GA is a robust method for problems involving subset-selection over a large search space, there are alternatives, most notably the Least Absolute Shrinkage And Selection Operator (LASSO) method (61). The presented approach can be interpreted as a heuristic direct search for the best-fit solution using the minimum number of non-zero regression coefficients (“best subset selection”), or an ℓ^0^-regularized optimization problem. The LASSO amounts to the relaxation to the best-fit solution with a minimum absolute-sum of regression coefficients, or an ℓ^1^-regularized optimization problem. While the discussions about the trade-offs between true best-subset and relaxed best-subset (LASSO) methods are available in the literature [see (62) for an exhaustive list of references], a comparison on this specific problem should be performed in future studies.

Our rationale for using a Bayesian logistic regression model along with feature selection through GA is to achieve a balance between computational efficiency and information obtained. The use of Bayesian inference provides the advantage of getting full posterior information. When compared with decision-tree-based prediction models such as classification and regression trees (CART), logistic regression model allows for a better interpretation of the effects of the individual predictor variables. It also offers a substantial computational advantage when there are a large number of predictors as in the present work.

Existing RA models primarily use genetic, environmental, and behavioral risk factors as predictors (35–37, 41). Karlson et al. reported a logistic regression model that uses a weighted GRS representing the aggregated effects of HLAs and SNPs associated with RA, age, sex, and smoking to predict RA that achieved an AUC of 0.660–0.752, depending on the dataset used (35). Subsequent works using the same model framework but including updated or additional predictor variables such as GRS incorporating newly validated RA risk alleles, exposure to silica, alcohol intake, education, parity, and some of the major interactions between predictors exhibited a similar classification performance (36, 37). A different model using genetic risk factors and smoking data, and determining risk through computer simulation and confidence interval based risk categorization achieved a higher discrimination ability of seropositive RA from control with AUC of 0.837–0.857, although the model is evaluated for male gender alone (41). Although genetic risk factors are demonstrated to be important in RA prediction in these models, our model does not include them considering the potential applicability in peripheral and rural health infrastructures where such advanced genotyping will unlikely be available for patients. Instead, common comorbidities and demographic variables, such as ethnicity, were incorporated in our model as predictors. The promise of our model in predicting RA is demonstrated by a high predictive accuracy in comparison to previous studies, especially when only a smaller subset of first-order variables are considered (see Supplementary Table 2). We speculate that a conflation of RA with other forms of arthritis in NHANES datasets could prevent our model achieving substantially higher predictive abilities after incorporation of higher-order effects. This conflation potentially results from self-reported diagnosis of RA and other arthritis in NHANES, and is reflected by a higher proportion of RA in the population than expected from the existing literature (5) (Supplementary Table 1).

Our results suggest that our model could achieve high predictive accuracy from the first-order variables alone when an appropriate set of risk factors are selected. While model predictive performance might not improve significantly by incorporating higher-order interactions in such a scenario, identifying the strong interactions could provide important clinical insight. Furthermore, in situations where health resources are highly constrained with severely limited data availability, higher-order interactions could play a significant role in achieving a sufficient degree of predictive accuracy. Our model could also be applicable to predict other chronic diseases that multiple, potentially interacting, factors are known to be associated with.

Even though NHANES provides a rich dataset of risk factors associated with RA, one limitation of the study comes from the self-reported nature of RA diagnosis, which tend to inflate the numbers through false positive diagnosis of other form of arthritis (63). Although a meta-analyses inferred that self-reported diagnosis is sufficiently accurate for large-scale epidemiological studies (64), the model could be made more robust by future validation and optimization with patient data where more rigorous criteria for RA diagnosis, such as the one provided by the American College of Rheumatology, is used (65). The ability to implement sample weights in the model could also marginally improve the model performance. The second limitation comes from the cross-sectional nature of the NHANES data, where the old and new RA cases cannot be discriminated. Furthermore, the comorbidities, socioeconomic and behavioral risk factors coexisted with RA in this data, and thus it could not be temporally resolved whether RA appeared before or after the manifestation of these risk factors. This restricts our model's prediction results on the NHANES dataset to be better interpreted as correlation rather than causation, essentially identifying risk factors and risk interactions associated with RA. We expect the model accuracy to improve along with the ability to infer a causal relationship by training with longitudinal data where the diagnosis of RA can be studied against a population with existing risk factors. Furthermore, Bayesian logistic regression model assumes a simple linear relationship between the predictors and the log-odds of having RA, however, the relationship could be more complex in reality. Although consideration of higher order interactions partially addresses this limitation, a better understanding of the relationship between risk factors and RA could help to construct a more accurate model in the future.

In summary, we have developed a model to predict RA from comorbidities, demographic, socioeconomic, and behavioral risk factors. The model demonstrated a high predictive accuracy in comparison with other models reported in the literature. Moreover, our model was able to identify important second- and third-order interactions between the risk factors, which may have important clinical relevance and stimulate further research to understand the mechanisms underlying such interactions. Since the model prediction utilizes patient information commonly available in a regular healthcare set-up, it has the future potential for translation to the clinical setting.

## Data Availability Statement

Publicly available datasets were analyzed in this study. This data can be found here: https://wwwn.cdc.gov/nchs/nhanes/.

## Author Contributions

LL, SM, and SS conceptualized the study. LL developed and executed the procedure. SM and MB verified the procedure. All authors contributed in writing the manuscript.

## Conflict of Interest

The authors declare that the research was conducted in the absence of any commercial or financial relationships that could be construed as a potential conflict of interest.

## Publisher's Note

All claims expressed in this article are solely those of the authors and do not necessarily represent those of their affiliated organizations, or those of the publisher, the editors and the reviewers. Any product that may be evaluated in this article, or claim that may be made by its manufacturer, is not guaranteed or endorsed by the publisher.

## Footnotes

^1^ https://www.cdc.gov/nchs/nhanes/

## Abbreviations

FAMD: factor analysis of mixed data
HDI: highest density interval
GA: genetic algorithm
SEC: socieconomic condition
IPR: income to poverty ratio
MA: mexican-american
OH: other hispanic
ONH: other non-hispanic
BP: systolic blood pressure
PHQ: patient health questionnaire.
