# Supplementary material for: A Bayesian Model to Analyze the Association of Rheumatoid Arthritis With Risk Factors and Their Interactions
Source: Front Public Health. 2021 Aug 16;9:693830. doi: 10.3389/fpubh.2021.693830 (PMC8415718; doi:10.3389/fpubh.2021.693830)
Supplement: Supplementary file 1 [file Data_Sheet_1.PDF]

Supplementary Information:A Bayesian Model to Analyze the Association of Rheumatoid Arthritis with Risk Factors and Their Interactions

LEON LUFKIN[1], MARKO BUDIŠIĆ[2], SUMONA MONDAL[2], AND SHANTANU SUR[3,*]

[1] *The Clarkson School, Clarkson University, Potsdam, NY 13699, US*
[2] *Department of Mathematics, Clarkson University, Potsdam, NY 13699, US*
[3] *Department of Biology, Clarkson University, Potsdam, NY 13699, US*
[*] *Correspondence: ssur@clarkson.edu*

1. SUPPLEMENTARY TABLES

	RA Prevalence	Proportion Male		RA Female-Male Ratio
		RA	No Arthritis	
Literature reported	~0.5–1%	~25%	~50%	~2–3
NHANES (before preprocessing)	3.01%	39.3%	49.6%	1.54
NHANES (after preprocessing)	6.55%	41.5%	52.7%	1.41

Table S1. RA prevalence, proportions of males in RA and no arthritis population, and female to male ratio in RA population are compared between literature reported values and NHANES data (before and after preprocessing).

Study	Dataset	Risk Factors	RA Type	AUC
Karlson, et. al. [1]	NHS*	Age, sex, geographic region, smoking	Seropositive	0.566
Karlson, et. al. [1]	EIRA	Age, sex, geographic region, smoking	Seropositive	0.626
Karlson, et. al. [1]	NHS	Age, sex, smoking + 8 HLA, 14 SNP alleles	Seropositive	0.660
Karlson, et. al. [1]	EIRA	Age, sex, smoking + 8 HLA, 14 SNP alleles	Seropositive	0.752
Chibnik, et. al. [2]	NHS	GRS, age, smoking	Seronegative	0.563
Chibnik, et. al. [2]	NHS	GRS, age, smoking	Seropositive	0.654
Chibnik, et. al. [2]	NHS	GRS, age, smoking	Erosive and seropositive	0.644
Chibnik, et. al. [2]	NHS	GRS, age, smoking	Erosive	0.712
Karlson, et. al. [3]	NHS	Age, smoking, alcohol, education, parity	Seropositive	0.655
Karlson, et. al. [3]	EIRA	Age, smoking, alcohol, education, parity	Seropositive & female†	0.632
Karlson, et. al. [3]	EIRA	Age, smoking, alcohol, education	Seropositive & male	0.685
This study	NHANES	Age, smoking	Self-reported	0.748
This study	NHANES	Age, sex, smoking	Self-reported	0.772

Table S2. Comparison of predictive ability of current study against from previous works. Abbreviations: NHS, US Nurses' Health Studies I & II; EIRA, Swedish Epidemiologic Investigation of RA; GRS, Genetic Risk Score. *All prediction using NHS was only performed on females. †"female" indicates prediction was only performed on female subjects; "male" indicates prediction was only performed on male subjects.

2. SUPPLEMENTARY FIGURE

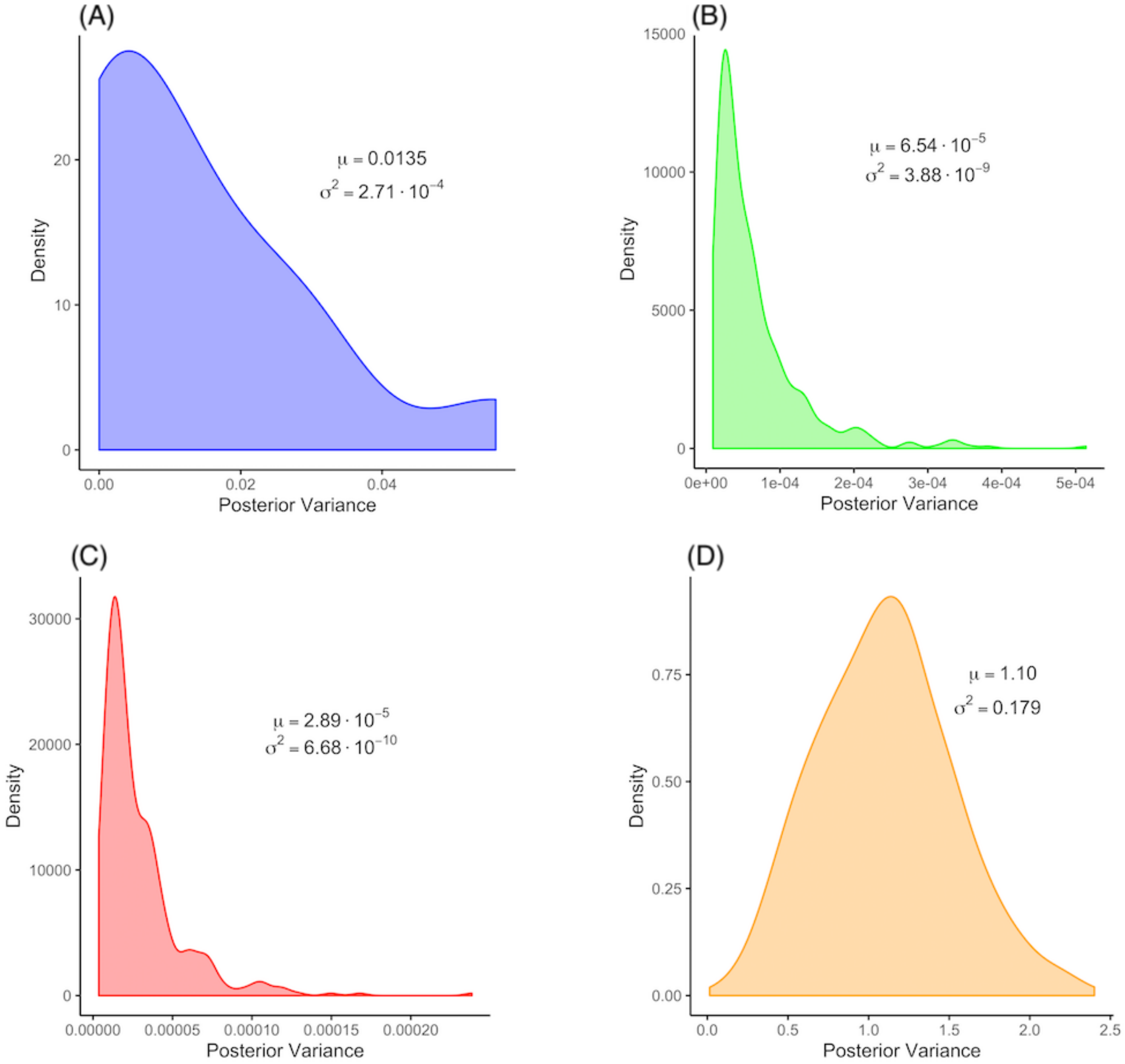

Figure S1. Distributions of posterior variances of estimated regression coefficients from four Bayesian approaches used to predict RA with the training dataset; mean and variance of distributions are shown. (**a**) Regression model using only the 14 first-order variables. (**b**) Estimates for all 489 variables using regression coefficients derived from 52 synthetic variables after FAMD. (**c**) Estimates for all 489 variables using regression coefficients derived from 33 synthetic variables selected by GA. (**d**) regression model using all 489 variables without FAMD.

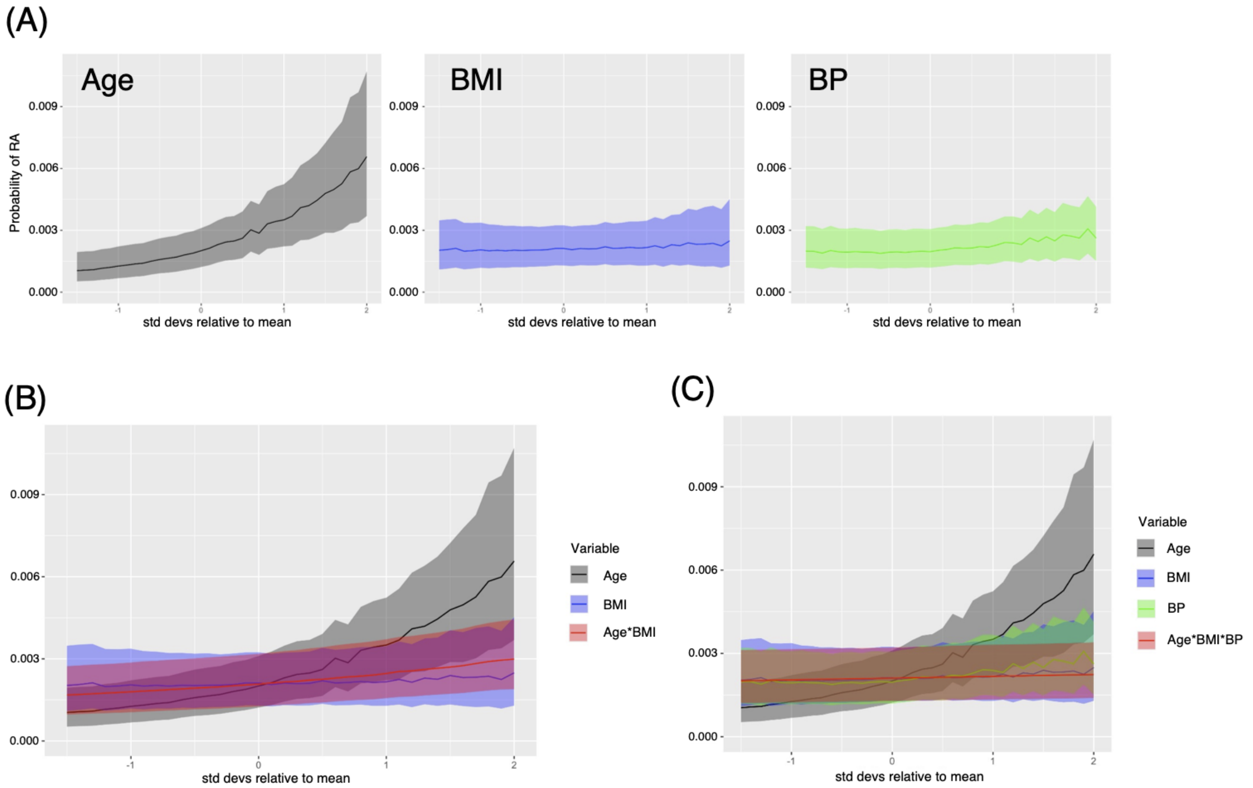

Figure S2. Marginal effects plots of (**A**) first-order variables age, BMI, and BP, (**B**) second-order interaction of age and BMI, and (**C**) third-order interaction of age, BMI, and BP, with first-order effects overlayed for comparison.

REFERENCES

1. E. W. Karlson, L. B. Chibnik, P. Kraft, J. Cui, B. T. Keenan, B. Ding, S. Raychaudhuri, L. Klareskog, L. Alfredsson, and R. M. Plenge, "Cumulative association of 22 genetic variants with seropositive rheumatoid arthritis risk," Annals rheumatic diseases **69**, 1077–1085 (2010).
2. L. B. Chibnik, B. T. Keenan, J. Cui, K. P. Liao, K. H. Costenbader, R. M. Plenge, and E. W. Karlson, "Genetic risk score predicting risk of rheumatoid arthritis phenotypes and age of symptom onset," PloS one **6**, e24380 (2011).
3. E. W. Karlson, B. Ding, B. T. Keenan, K. Liao, K. H. Costenbader, L. Klareskog, L. Alfredsson, and L. B. Chibnik, "Association of environmental and genetic factors and gene–environment interactions with risk of developing rheumatoid arthritis," Arthritis care & research **65**, 1147–1156 (2013).
